# Surgical aspects of pneumatosis cystoides intestinalis: two case reports

**DOI:** 10.4076/1757-1626-2-6452

**Published:** 2009-08-12

**Authors:** Engelbert Schröpfer, Thomas Meyer

**Affiliations:** Department of General, Visceral, Vascular and Transplant-Surgery, Julius-Maximilians-University of WürzburgWürzburgGermany

## Abstract

**Introduction:**

Pneumatosis cystoides intestinalis is a rare disease usually caused by an underlying condition. It is defined as air filled cysts within the wall of the gastrointestinal tract. The purpose of this paper is the development of an algorithm for the surgical therapy of PCI based one two case reports.

**Case presentations:**

A 17-year-old girl with Down syndrome and leucopenia due to chemotherapy for acute lymphatic leukemia was admitted with acute septic conditions and PCI. Explorative laparotomy revealed acute ischemia of the right colon and resection of the affected intestine was performed. After a short interval in the intensive care unit the patient was referred to the pediatric department. The second patient, a 79 year old man, with urothelial carcinoma and carcinoma of the prostate presented a distended abdomen. CT-scan revealed PCI and adhesive strangulation of intestines. Therefore only adhesiolysis was performed and PCI was treated conservatively.

**Conclusion:**

Only patients with increased inflammatory parameters in laboratory findings or signs of sepsis, peritonitis or bowel perforation in combination with PCI should receive an explorative laparotomy.

## Introduction

Pneumatosis cystoides intestinalis (PCI) is defined as air filled cysts within the wall of the gastrointestinal tract. Duvernoi first documented PCI in his manuscript “Aer intestinorum tam sub extima quam intima tunica inclussus”, published 1730 [1]. In 1876, Bang described the histomorphologic changes caused by PCI [2] and in 1899 Hahn described PCI living patients [3]. The first preoperative diagnosis of PCI through radiologic findings was made by Baumann-Schenker 1939 [4].

The incidence of PCI is unknown; the frequency is reported to be highest in the sixth decade. Primary PCI is extremely rare, in most cases PCI is due to an underlying disease or condition (traumatic and mechanical, inflammatory and autoimmune diseases, drug induced, immunosuppression, transplantation or neoplasm) [5]. In a review of 213 pathologic specimens, Koss noted 1952 that 85% of PCI are attributed to a secondary disease process [6]. All parts of the gastrointestinal tract may be affected; the small bowel is involved in 42%, the colon in 36% or both in 22% of the cases [7]. The purpose of this paper is the development of an algorithm for the surgical therapy of PCI based one two case reports.

## Case presentations

### Case report 1

The first case reports on a 17 year old white European girl, with Down syndrome, steroid induced diabetes and relapsed acute lymphatic leukemia (ALL) since four months. She was admitted to our surgical department from the pediatric intensive ward for further treatment of progressive sepsis and free abdominal air. Because of chemotherapy for ALL, the patient was in pancytopenia, with white blood cell count at 100 per µl [5.000 - 10.000 per µl], platelets at 28.000/µl [150.000 - 450.000 per µl] and Hb at 7.6 g/dl [12-16 g/dl]. Due to a CRP concentration of 18.98 mg/dl [0-0.5 mg/dl], an antibiotic treatment with Ceftazidim, Gentamycin and Teicoplanin had already been started on the pediatric intensive care ward. Blood culture test showed presence of beta lactam resistant Escherichia coli, therefore Meropenem was added. Abdominal sonography revealed free abdominal air near the portal vein.

**Figure 1A and B.**
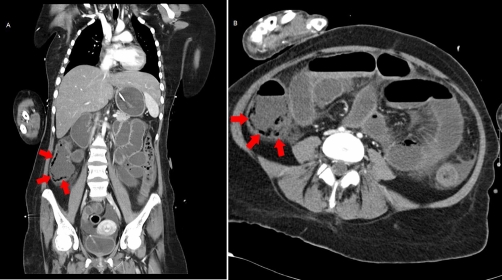
Abdominal and thoracic computer tomography of patient 1 coronal view: Multiple air filled cysts in the intestine wall along the right hemicolon can be seen (red arrows). Diagnosis Pneumatosis cystoides intestinalis was made. The sonographic findings (free abdominal air near the portal vein) were not confirmed in CT.

Cardiopulmonary resuscitation was performed on the pediatric intensive care ward due to acute circulatory failure. After resuscitation abdominal computer tomography (CT) was carried out and multiple air filled cysts within the cecal wall could be seen (Figure 1A and B). The sonographic finding of free air near the portal vein was confirmed in CT. The decision for immediate laparotomy under emergency conditions was made based on the radiologic findings.

**Figure 2.**
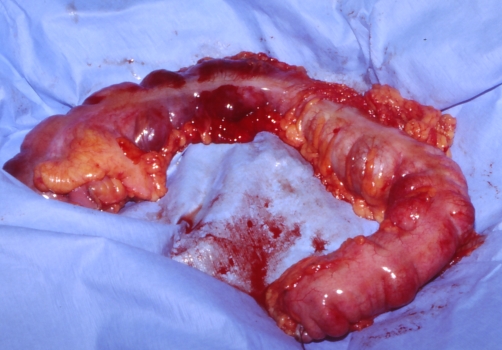
Photography during operation of patient 1: View of the colon from the ceacum to the descending colon with livid discoloration of the right hemicolon and the transverse colon as a sign of acute ischemia.

During surgery acute ischemia of the right hemicolon and the transverse colon could be seen (Figure 2). Resection of the right ischemic hemicolon was performed and because of the aplasia and sepsis, we decided on closure of the colon-stump and ileostomy. Fresh frozen plasma, red blood cell concentrates and platelet concentrates were given postoperative and antibiotic treatment was changed to Meropenem, Vancomycin, Ciprofloxacin, Metronidazol and Fluconazol. Patient was isolated and treatment with granulocyte-colony stimulating factor (GCSF) was started. On recovery from the septis, catecholamine treatment was reduced and after extubation patient was referred to the pediatric department for continuing chemotherapy.

Pathological findings of the resected tissue revealed an acute hemorrhagic infarction of the colon with necrosis of the mucosa and ectasia of the submucosal and subserosal vessels, without proof of thromboses. No bacteria and no malignancy were found.

### Case report 2

A 79 year old, white European male patient suffering from an urothelial carcinoma of the bladder and carcinoma of the prostate was treated in the department of urology. Radical cysto-prostatectomy (RO) was performed in January 2008. Two days after the operation, an acute ischemia of the right leg occurred and angiography revealed a complete obstruction of the right common iliac artery. A femoro-fermoral cross-over bypass was performed by the department of vascular surgery. Additionally, due to a beginning compartment syndrome of the right leg, fasciotomy was performed. After several debridements of the fasciotomy-wound and vacuum-assisted closure of the wound, mesh skin grafting was carried out.

**Figure 3.**
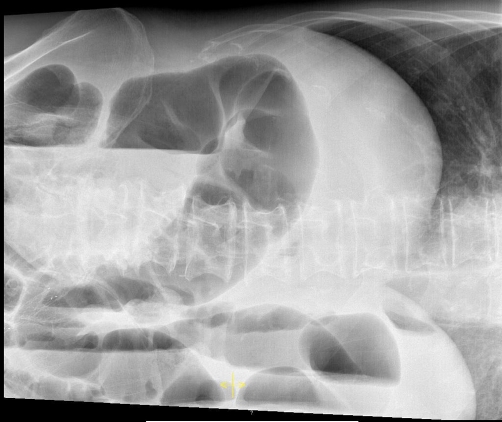
Plain abdominal radiography of patient 2: Distension of the large bowel and gas-fluid-levels as signs for a large bowel obstruction.

**Figure 4A and B.**
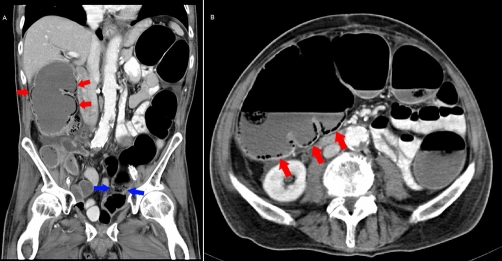
Abdominal computer tomography of patient 2 coronal view: Multiple air filled cysts in the intestine wall along the right hemicolon and a massive dilatation of the whole colon can be seen. Diagnosis pneumatosis cystoides intestinalis (red arrows) and ileus of the colon was made. The suspected level of obstruction is indicated with blue arrows.

One month after cysto-prostatectomy the patient showed signs of an acute abdomen with progressive abdominal pain and guarding. His white blood cell count was 14.400 per μl and the CRP concentration was 4.75 mg/dl [0-0.5 mg/dl]. Lactate measurement was 1.6 mmol/l [0.7 - 2.1 mmol/l]. Because plain radiography showed signs of large bowel obstruction (Figure 3), CT was carried out. Abdominal CT revealed large bowel obstruction and air filled cysts in the whole colonic wall. (Figure 4A and B). The suspected diagnosis of adhesive bowel obstruction was made. Due to the radiologic findings and clinical signs of an acute abdomen explorative laparotomy and adhesiolysis were performed. Two months after admittance the patient was discharged into rehabilitation.

## Discussion

PCI is a rare disease usually caused by an underlying condition. The incidence is unknown due to the asymptomatic course of the disease in most cases. Primary PCI is extremely rare, Shawn et al. divided the underlying conditions for secondary PCI into six groups, traumatic and mechanical, inflammatory and autoimmune, infectious, pulmonary, drug induced and other [8]. The most common symptoms of PCI are diarrhea or constipation, bloody stools, abdominal pain, flatus or weight loss. It has been suggested that these symptoms are caused by mechanical effects of the cysts, but in many cases even large cysts remain asymptomatic; thus showing no correlation between clinical manifestation and the amount of intramural gas [9]. Approximately 3% of afflicted patients experience a complication, such as pneumoperitoneum, volvolus, intestinal obstruction intussusception, hemorrhage or perforation.

The pathogenesis of PCI has been discussed for a long time and multiple theories exist. Three different possibilities for the source of gas within the intestine wall have been considered: intraluminal gas, pulmonary gas and gas produced by bacteria. Two basic mechanical features are responsible for intrusion of intraluminal gas into the bowel wall: mucosal injury, the most important and prevalent feature, and increased intralumenal pressure; or both. Increased intralumenal pressure may be produced by bowel obstruction, ileus, or iatrogenically by upper or lower GI endoscopy. Damages in the mucosa may result from an inflammatory process, a defect in the gut immune barrier and steroid or cytotoxic medical therapy.

The possibility of pulmonary gas as a source for PCI is based on the theory of air migration along vessels within the mediastinum, retroperitoneum and mesentery after alveolar rupture in pulmonary diseases. The absence of interstitial emphysema within the lung or the mesentery in many of the cases has led to contrasting opinions regarding this theory [10].

The disappearance of PCI during antimicrobial treatment in some cases has led to the theory that gas producing bacteria is reasonable for PCI [9]. Direct invasion of the wall and alteration of the intraluminal gas content enables bacteria to form intramural gas. Mucosal damage and immune deficiency leads to bacterial invasion of intramural compartments. However, there is a lack of evidence for bacteria within the cysts themselves [7]. Direct gas diffusion across the mucosa is caused by a gradient between intraluminal and serum partial pressures. Bacteria produce intraluminal hydrogen tension higher than nitrogen tension in blood leading to hydrogen diffusion towards the submucosal vessels, where it is followed by nitrogen, oxygen and carbon dioxide from circulation [11].

Plain radiography, barium enema, ultrasound, abdominal CT and endoscopy can be helpful in diagnosing PCI, CT being the best method with the highest sensitivity. According to Jamart, PCI can be detected in approximately two thirds of patients with plain radiographic findings [12]. Many authors describe the value of ultrasound for diagnosing PCI and portal venous air [13]. Barium enema has been used but the findings can be confused with polyposis as they have similar appearances [14]. During endoscopy macroscopic elevations of the mucosa can be seen and some authors recommend puncture to confirm the diagnosis [7]. But removing of a polyp that in fact was a cyst has caused perforation of the bowel in some cases [15]. Computed tomography can distinguish PCI from intraluminal air or submucosal fat and provides survey of the abdomen for diagnosis of associated pathological conditions.

Patients with a radiographic diagnosis of PCI should have a thorough history and physical examination and only along with the knowledge of the underlying disease the decision for an explorative laparotomy can be made. PCI can be managed conservatively in most cases [5,8]. The major problem for surgeons is identifing the patients requiring surgical intervention. Laparotomy should not be performed based on radiographic findings alone. Good results with inspired oxygen have been reported in PCI with underlying diseases that do not require surgery [16]. Pneumoperitoneum is not necessarily caused by bowel perforation but may represent a ruptured cyst [17]. Gas in the portal system is associated with a high mortality rate of 37% and often occurs with ischemic bowel [18].

**Figure 5.**
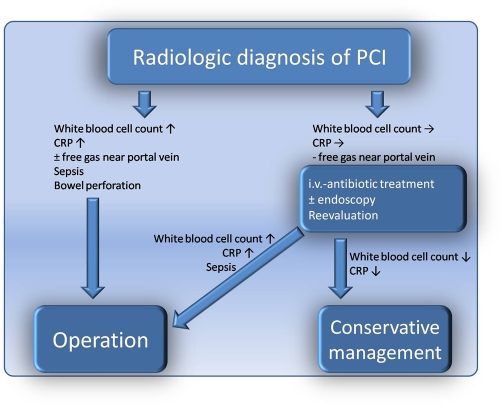
Algorithm of surgical management of PCI.

In case of a radiologic diagnosis of PCI in a patient, we recommend a standardized procedure (Figure 5). With laboratory findings of elevated CRP or white blood cell count, as well as signs of sepsis, bowel perforation or free gas near the portal vein immediate surgery is indicated. In patients with normal or slightly increased inflammatory parameters in blood samples and no signs of sepsis, bowel perforation or free gas, we recommend modest therapy comprising of i.v. antibiotics and, optionally, endoscopy for further clarification as well as reevaluation after a definite time interval (12-24h). Conservative management is preferable, but in the case of increasing CRP or white blood cell surgery should be performed.

Our first case demonstrates that PCI in radiographic findings in combination with sepsis or signs of infection is always an indication for explorative laparotomy. PCI was likely caused by bacterial invasion of the intestine wall because of immune deficiency. The positive testing for Escherichia coli in blood culture confirmed this suspicion. The second patient underwent a laparotomy because of ileus. An operation wouldn’t have been necessary based on the PCI alone, which was in our opinion a consequence of the increased intralumenal pressure.

## Conclusion

PCI as a radiographic finding does not routinely require laparotomy. A thorough history and physical examination are obligatory since PCI is mostly a consequence of an underlying disease. PCI can be managed conservatively in most cases. A lapartomy without clear indication might even worsen the general condition of the patient. Patients with increased inflammatory parameters in laboratory findings or signs of sepsis, peritonitis or bowel perforation in combination with PCI should receive an explorative laparotomy.

## Abbreviations

ALL: acute lymphatic leukemia
CRP: C-reactive protein
CT: Computed tomography
GCSF: granulocyte-colony stimulating factor
PCI: Pneumatosis cystoides intestinalis
